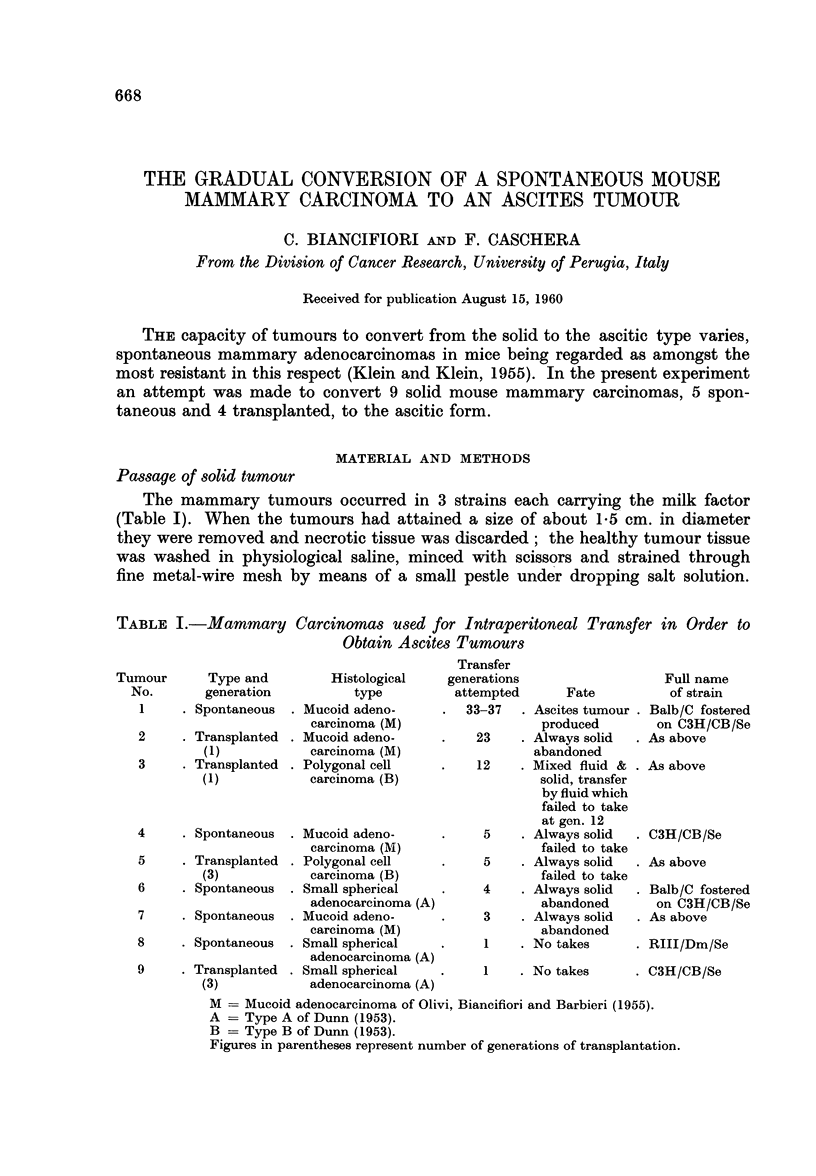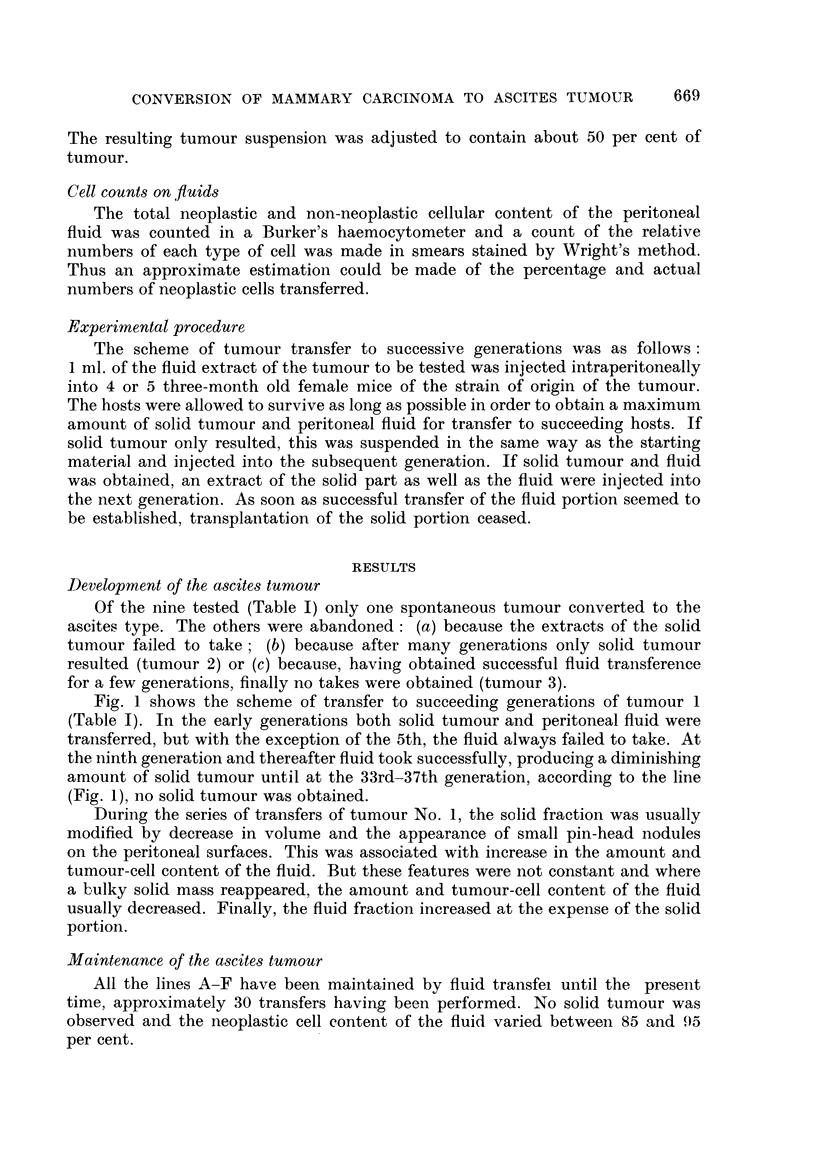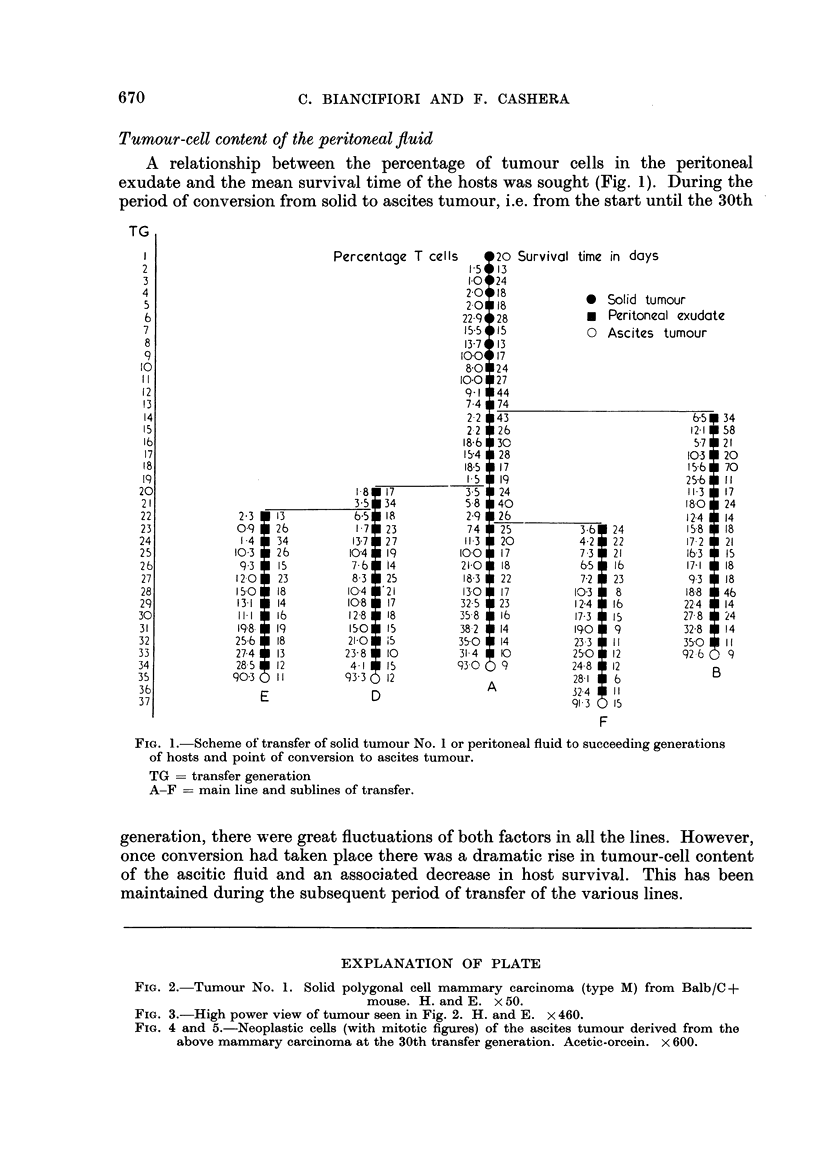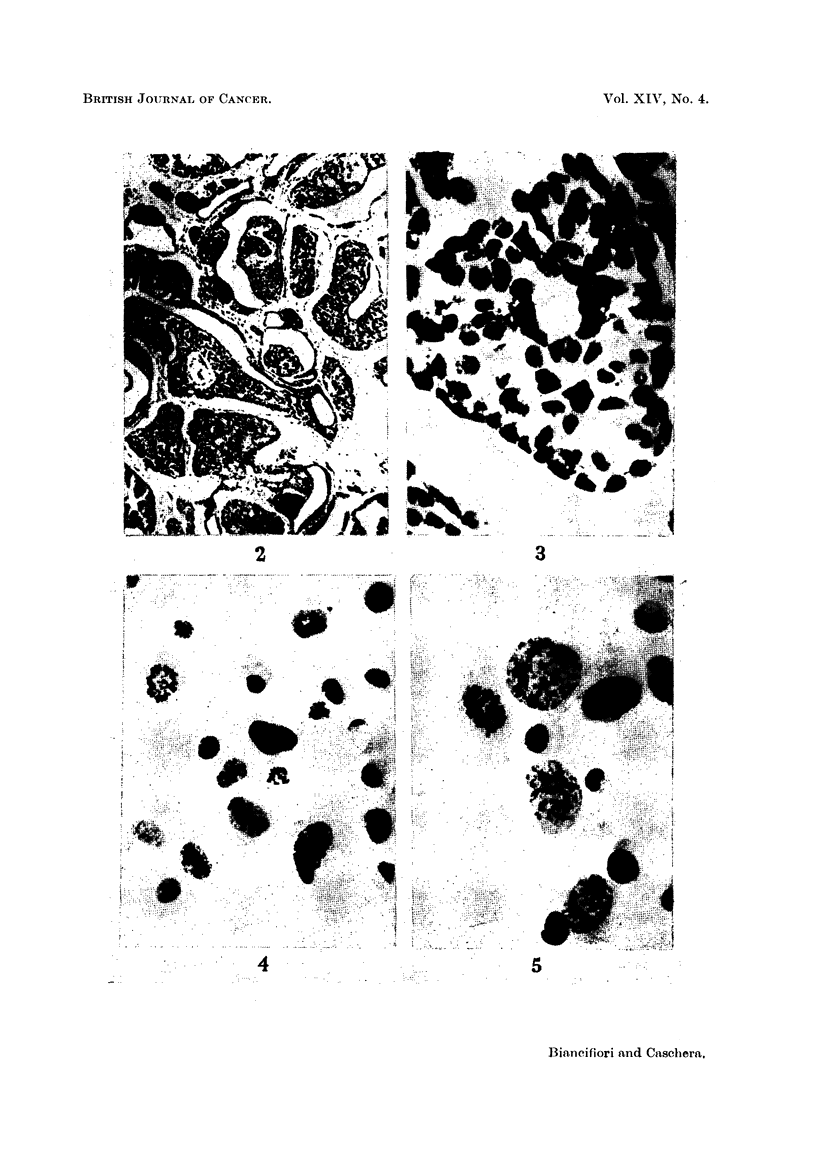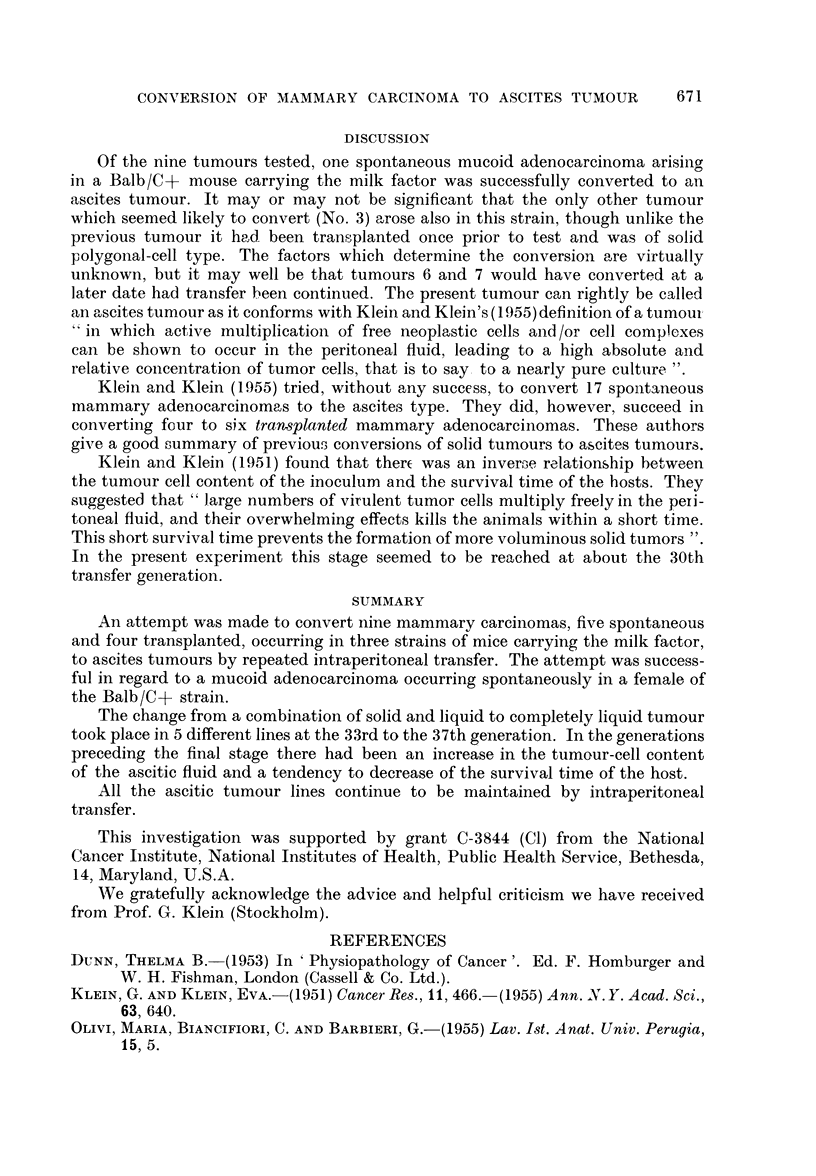# The Gradual Conversion of a Spontaneous Mouse Mammary Carcinoma to an Ascites Tumour

**DOI:** 10.1038/bjc.1960.74

**Published:** 1960-12

**Authors:** C. Biancifiori, F. Caschera

## Abstract

**Images:**


### THE GRADUAL CONVERSION OF A SPONTANEOUS MOUSE MAMMARY CARCINOMA TO AN ASCITES TUMOUR

C. BIANCIFIORI AND F. CASCHERA

*From the Division of Cancer Research, University of Perugia, Italy*

Received for publication August 15, 1960

THE capacity of tumours to convert from the solid to the ascitic type varies, spontaneous mammary adenocarcinomas in mice being regarded as amongst the most resistant in this respect (Klein and Klein, 1955). In the present experiment an attempt was made to convert 9 solid mouse mammary carcinomas, 5 spontaneous and 4 transplanted, to the ascitic form.

### MATERIAL AND METHODS

*Passage of solid tumour*

The mammary tumours occurred in 3 strains each carrying the milk factor (Table I). When the tumours had attained a size of about 1·5 cm. in diameter they were removed and necrotic tissue was discarded ; the healthy tumour tissue was washed in physiological saline, minced with scissors and strained through fine metal-wire mesh by means of a small pestle under dropping salt solution.

TABLE I.—*Mammary Carcinomas used for Intraperitoneal Transfer in Order to Obtain Ascites Tumours*

| Tumour No. | Type and generation | Histological type | Transfer generations attempted | Fate | Full name of strain |
|---|---|---|---|---|---|
| 1 | Spontaneous | Mucoid adenocarcinoma (M) | 33–37 | Ascites tumour produced | Balb/C fostered on C3H/CB/Se |
| 2 | Transplanted (1) | Mucoid adenocarcinoma (M) | 23 | Always solid abandoned | As above |
| 3 | Transplanted (1) | Polygonal cell carcinoma (B) | 12 | Mixed fluid & solid, transfer by fluid which failed to take at gen. 12 | As above |
| 4 | Spontaneous | Mucoid adenocarcinoma (M) | 5 | Always solid failed to take | C3H/CB/Se |
| 5 | Transplanted (3) | Polygonal cell carcinoma (B) | 5 | Always solid failed to take | As above |
| 6 | Spontaneous | Small spherical adenocarcinoma (A) | 4 | Always solid abandoned | Balb/C fostered on C3H/CB/Se |
| 7 | Spontaneous | Mucoid adenocarcinoma (M) | 3 | Always solid abandoned | As above |
| 8 | Spontaneous | Small spherical adenocarcinoma (A) | 1 | No takes | RIII/Dm/Se |
| 9 | Transplanted (3) | Small spherical adenocarcinoma (A) | 1 | No takes | C3H/CB/Se |

M = Mucoid adenocarcinoma of Olivi, Biancifiori and Barbieri (1955).
A = Type A of Dunn (1953).
B = Type B of Dunn (1953).
Figures in parentheses represent number of generations of transplantation.

The resulting tumour suspension was adjusted to contain about 50 per cent of tumour.

*Cell counts on fluids*

The total neoplastic and non-neoplastic cellular content of the peritoneal fluid was counted in a Burker's haemocytometer and a count of the relative numbers of each type of cell was made in smears stained by Wright's method. Thus an approximate estimation could be made of the percentage and actual numbers of neoplastic cells transferred.

*Experimental procedure*

The scheme of tumour transfer to successive generations was as follows : 1 ml. of the fluid extract of the tumour to be tested was injected intraperitoneally into 4 or 5 three-month old female mice of the strain of origin of the tumour. The hosts were allowed to survive as long as possible in order to obtain a maximum amount of solid tumour and peritoneal fluid for transfer to succeeding hosts. If solid tumour only resulted, this was suspended in the same way as the starting material and injected into the subsequent generation. If solid tumour and fluid was obtained, an extract of the solid part as well as the fluid were injected into the next generation. As soon as successful transfer of the fluid portion seemed to be established, transplantation of the solid portion ceased.

### RESULTS

*Development of the ascites tumour*

Of the nine tested (Table I) only one spontaneous tumour converted to the ascites type. The others were abandoned : (*a*) because the extracts of the solid tumour failed to take ; (*b*) because after many generations only solid tumour resulted (tumour 2) or (*c*) because, having obtained successful fluid transference for a few generations, finally no takes were obtained (tumour 3).

Fig. 1 shows the scheme of transfer to succeeding generations of tumour 1 (Table I). In the early generations both solid tumour and peritoneal fluid were transferred, but with the exception of the 5th, the fluid always failed to take. At the ninth generation and thereafter fluid took successfully, producing a diminishing amount of solid tumour until at the 33rd–37th generation, according to the line (Fig. 1), no solid tumour was obtained.

During the series of transfers of tumour No. 1, the solid fraction was usually modified by decrease in volume and the appearance of small pin-head nodules on the peritoneal surfaces. This was associated with increase in the amount and tumour-cell content of the fluid. But these features were not constant and where a bulky solid mass reappeared, the amount and tumour-cell content of the fluid usually decreased. Finally, the fluid fraction increased at the expense of the solid portion.

*Maintenance of the ascites tumour*

All the lines A–F have been maintained by fluid transfer until the present time, approximately 30 transfers having been performed. No solid tumour was observed and the neoplastic cell content of the fluid varied between 85 and 95 per cent.

*Tumour-cell content of the peritoneal fluid*

A relationship between the percentage of tumour cells in the peritoneal exudate and the mean survival time of the hosts was sought (Fig. 1). During the period of conversion from solid to ascites tumour, i.e. from the start until the 30th

FIG. 1.—Scheme of transfer of solid tumour No. 1 or peritoneal fluid to succeeding generations of hosts and point of conversion to ascites tumour.

TG = transfer generation

A–F = main line and sublines of transfer.

generation, there were great fluctuations of both factors in all the lines. However, once conversion had taken place there was a dramatic rise in tumour-cell content of the ascitic fluid and an associated decrease in host survival. This has been maintained during the subsequent period of transfer of the various lines.

---

### EXPLANATION OF PLATE

FIG. 2.—Tumour No. 1. Solid polygonal cell mammary carcinoma (type M) from Balb/C+ mouse. H. and E. ×50.

FIG. 3.—High power view of tumour seen in Fig. 2. H. and E. ×460.

FIG. 4 and 5.—Neoplastic cells (with mitotic figures) of the ascites tumour derived from the above mammary carcinoma at the 30th transfer generation. Acetic-orcein. ×600.

2

3

4

5

### DISCUSSION

Of the nine tumours tested, one spontaneous mucoid adenocarcinoma arising in a Balb/C+ mouse carrying the milk factor was successfully converted to an ascites tumour. It may or may not be significant that the only other tumour which seemed likely to convert (No. 3) arose also in this strain, though unlike the previous tumour it had been transplanted once prior to test and was of solid polygonal-cell type. The factors which determine the conversion are virtually unknown, but it may well be that tumours 6 and 7 would have converted at a later date had transfer been continued. The present tumour can rightly be called an ascites tumour as it conforms with Klein and Klein's (1955) definition of a tumour "in which active multiplication of free neoplastic cells and/or cell complexes can be shown to occur in the peritoneal fluid, leading to a high absolute and relative concentration of tumor cells, that is to say to a nearly pure culture".

Klein and Klein (1955) tried, without any success, to convert 17 spontaneous mammary adenocarcinomas to the ascites type. They did, however, succeed in converting four to six *transplanted* mammary adenocarcinomas. These authors give a good summary of previous conversions of solid tumours to ascites tumours.

Klein and Klein (1951) found that there was an inverse relationship between the tumour cell content of the inoculum and the survival time of the hosts. They suggested that "large numbers of virulent tumor cells multiply freely in the peritoneal fluid, and their overwhelming effects kills the animals within a short time. This short survival time prevents the formation of more voluminous solid tumors". In the present experiment this stage seemed to be reached at about the 30th transfer generation.

### SUMMARY

An attempt was made to convert nine mammary carcinomas, five spontaneous and four transplanted, occurring in three strains of mice carrying the milk factor, to ascites tumours by repeated intraperitoneal transfer. The attempt was successful in regard to a mucoid adenocarcinoma occurring spontaneously in a female of the Balb/C+ strain.

The change from a combination of solid and liquid to completely liquid tumour took place in 5 different lines at the 33rd to the 37th generation. In the generations preceding the final stage there had been an increase in the tumour-cell content of the ascitic fluid and a tendency to decrease of the survival time of the host.

All the ascitic tumour lines continue to be maintained by intraperitoneal transfer.

This investigation was supported by grant C-3844 (Cl) from the National Cancer Institute, National Institutes of Health, Public Health Service, Bethesda, 14, Maryland, U.S.A.

We gratefully acknowledge the advice and helpful criticism we have received from Prof. G. Klein (Stockholm).

### REFERENCES

DUNN, THELMA B.—(1953) In 'Physiopathology of Cancer'. Ed. F. Homburger and W. H. Fishman, London (Cassell & Co. Ltd.).

KLEIN, G. AND KLEIN, EVA.—(1951) *Cancer Res.*, **11**, 466.—(1955) *Ann. N.Y. Acad. Sci.*, **63**, 640.

OLIVI, MARIA, BIANCIFIORI, C. AND BARBIERI, G.—(1955) *Lav. Ist. Anat. Univ. Perugia*, **15**, 5.